# May–June Maximum Temperature Reconstruction from Mean Earlywood Density in North Central China and Its Linkages to the Summer Monsoon Activities

**DOI:** 10.1371/journal.pone.0107501

**Published:** 2014-09-10

**Authors:** Feng Chen, Yujiang Yuan

**Affiliations:** Key Laboratory of Tree-Ring Physical and Chemical Research of China Meteorological Administration/Xinjiang Laboratory of Tree-Ring Ecology, Institute of Desert Meteorology, China Meteorological Administration, Urumqi, Xinjiang, China; University of California at San Diego, United States of America

## Abstract

Cores of *Pinus tabulaformis* from Tianshui were subjected to densitometric analysis to obtain mean earlywood density data. Climate response analysis indicates that May–June maximum temperature is the main factor limiting the mean earlywood density (EWD) of Chinese pine trees in the Shimen Mountains. Based on the EWD chronology, we have reconstructed May–June maximum temperature 1666 to 2008 for Tianshui, north central China. The reconstruction explains 40.1% of the actual temperature variance during the common period 1953–2008. The temperature reconstruction is representative of temperature conditions over a large area to the southeast and northwest of the sampling site. Preliminary analysis of links between large-scale climatic variation and the temperature reconstruction shows that there is a relationship between extremes in spring temperature and anomalous atmospheric circulation in the region. It is thus revealed that the mean earlywood density chronology of *Pinus tabulaformis* has enough potential to reconstruct the temperature variability further into the past.

## Introduction

The meteorological station records in most parts of the world show that temperatures have been increasing over the 20th century, and this global warming has been paid much attention to by scientists and the public [1]. It is therefore important to develop reconstructions on time-scales of centuries to millennia of temperature variability in different areas in order to gain further understanding of 20th century warming. A number of such studies have been recently carried out in the Northern Hemisphere providing the opportunity of recognizing previous climatic changes in various regions [2]–[15]. However, to further improve the spatial and temporal coverage of tree-ring network in Asia, more tree-ring chronologies are still needed.

Because of their high correlation, high resolution, and reliability, latewood densities (maximum density and mean latewood density) from the high latitude and altitude areas of the Northern Hemisphere are widely used for the purpose of reconstructing previous warm season temperature regimes [4], [13], [16],[17]. The high temperature during the growing season may play a very important role in accumulating wood density during the radial developing process of tree growth under cold and wet environment. Particularly in the later part of growing season (July to September), maximum density had a remarkable correlation with mean maximum temperature. Even though wood tracheid development and photosynthetic accumulation occur throughout the growing season, from the physiological perspective, cambium division and lengthening occurred mainly in the early part of growing season, as shown by enlarging tree radials. In contrast, wood cell was thickening mostly in the later part of the growing season [18], [19]. However, in north central China, it is difficult to find high correlations of latewood densities with temperature. At the same time, due to typical monsoon climate, these areas are often threatened by drought, and the drought is a major cause of the “divergence problem” [20]. Thus, we need to find other new proxies to reconstruct temperature.

In this paper, we developed a mean earlywood density (EWD) chronology from tree rings of Chinese pine in the Shimen Mountains, Tianshui, north central China. The goals of this study were: (1) to investigate the climatic response of the new chronology and reconstruct spring (May to June) maximum temperature history of Tianshui since AD 1666, (2) to disclose the spatial representation of the new reconstruction and (3) to reveal the linkages between the Asian summer monsoon.

## Data and Methods

### Study area and tree-ring material

**Figure 1:**
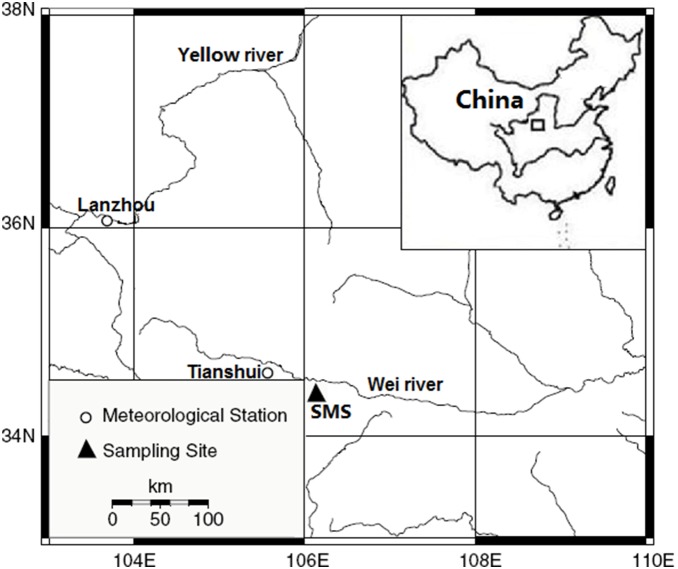
Location map of sampling site and meteorological stations.

The study area is located in the Shimen Mountains (SMS, 34°27′N, 106°09′E), which belongs to the transition zone between the Loess Plateau and the Qinling Mountains (**Figure 1**). The mountain stretches about 30 km from northeast to southwest. The highest peak rises to 2100 m a.s.l. This region is characterized as warm temperate semi-humid monsoon climate. According to the records from the nearest meteorological station (Tianshui) which is about 35 km northwest of the sampling site, the annual mean temperature is 11.0°C, and the annual mean total precipitation and evaporation is about 521.0 mm and 1910.4 mm, respectively. The maximum monthly temperature is seen in July (22.8°C), and the minimum monthly temperature in January (−2.2°C). The maximum monthly precipitation is found in July (92.3 mm), and the minimum precipitation in December (3.2 mm). Precipitation during the monsoonal season (June–September) accounts for 64.8% of the total annual precipitation.

The tree-ring samples were obtained from a northwest-facing slope of the mountain, an area of sparsely distributed and well preserved old forests. The study was also approved by the Gansu Forestry Department. Broad-leaved tree species found in valleys are replaced by pine forests in the peak area. Chinese pine (*Pinus tabulaformis*) is the dominant tree species, which is also an endemic tree species in north China. In this open-canopy site, 38 cores (10 mm) and 25 cores (5 mm) were taken at breast height from 25 Chinese pines along an altitudinal gradient from 2050 m to 2100 m. All the trees from which core samples were taken grew in rocky and shallow soil with a slope inclination from 10 to 35°.

### Chronology development

The cores were mounted and prepared following standard procedures [21], [22]. The measurement of tree-ring widths of all cores was taken using a Velmex measuring system (Velmex Inc., Bloomfield, New York). After obtaining the tree-ring width measurements, the pine resin of the 10 mm cores was extracted with hot water (at 80°C) and 95% alcohol for 96 hours. After air-drying, the cores were cut transversely into strips of 1±0.02 mm in thickness with a twin bladed saw (DENDRO-CUT2003). The strips were subjected to X-ray analysis. These X-ray radiographs were scanned on the DENDRO2003 tree-ring workstation to obtain mean earlywood density (EWD). To obtain good measurements, the steps described by Schweingruber et al. [23] were adopted. The boundary between earlywood and latewood was set for each ring at 50% of the difference between maximum and minimum density.

The cross-dating of tree-ring widths was checked using the COFECHA program first [24]. EWD data was compared to the tree-ring width cross-matching results. The mean earlywood density chronology building was performed using the ARSTAN program [25]. The negative exponential curve was fitted to each of time-series of measurement to remove the age-related non-climatic trends. The detrended individual ring-width series were then averaged using a biweight robust mean function to create a standard EWD chronology.

In order to estimate the reliability of the EWD chronology, the expressed population signal (EPS) and *R*_bar_ values were computed over gliding windows separated by 30 years. EPS evaluates the relationship between the sample size of a chronology and the common variance or “signal” within a chronology [26]. A threshold of EPS>0.80 was employed to determine the most reliable period of the chronology. *R*_bar_ is the average of all pairwise correlations for detrended tree-ring series within a chronology.

### Climate data and statistical analysis

**Figure 2:**
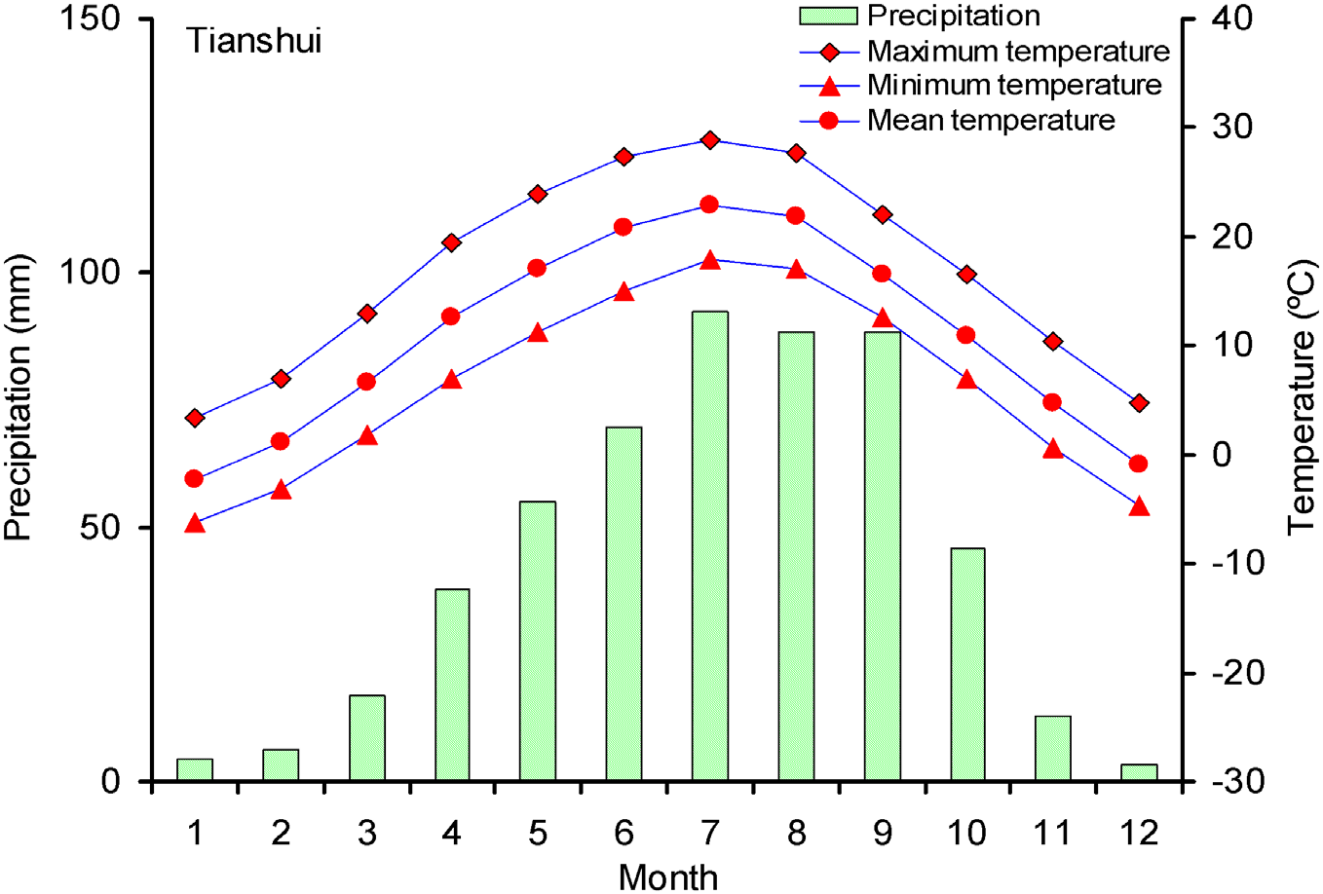
Monthly temperatures (°C) and precipitation (mm) in Tianshui for the period 1953 to 2008.

Climate data (**Figure 2**), including monthly precipitation and (maximum-T_max_, mean-T_mean_, minimum-T_min_) temperature from the Tianshui meteorological station (34°35′N, 105°45′E, 1142.6 m a.s.l., 1953–2008) were selected to analyze the growth-climate relationship. The bootstrapped response and correlation function analysis was done using the EWD chronology and climate data covering the same period to determine the most appropriate model for reconstruction [27]. All statistical procedures were evaluated at *P*<0.05 level of significance using the soft DENDROCLIM2002 [28]. These analyses used a 14-month climate window extending from July of the prior year to September of the growth year.

A linear regression equation between the predictor (EWD index) and the predictand (temperature) was computed for the calibration period [29]. The leave-one-out cross-validation method was further employed to evaluate the statistical fidelity of our reconstruction model [30]. The testing statistics used included the reduction of error (RE), the product means test, the sign test, and the Pearson’s correlation coefficient [29].

To demonstrate our record’s geographical representation, we conducted spatial correlations between our temperature reconstruction and the gridded temperature dataset of CRU TS 3.1 [31]. However, weather stations were not installed in many regions of China before 1950s. Thus, we conducted spatial correlations for the period 1953–2008. Correlations were calculated after removing the linear trends of data, by using the detrending option of the KNMI Climate Explorer (http://climexp.knmi.nl). Composites of April–June 850-hPa vector wind anomalies from the 1981–2010 mean were created using National Centers for Environmental Prediction–National Center for Atmospheric Research reanalysis data [32] for the highest and lowest deciles of reconstructed spring temperature in the period 1948–2010.

## Results and Discussion

### Temperature reconstruction

**Figure 3:**
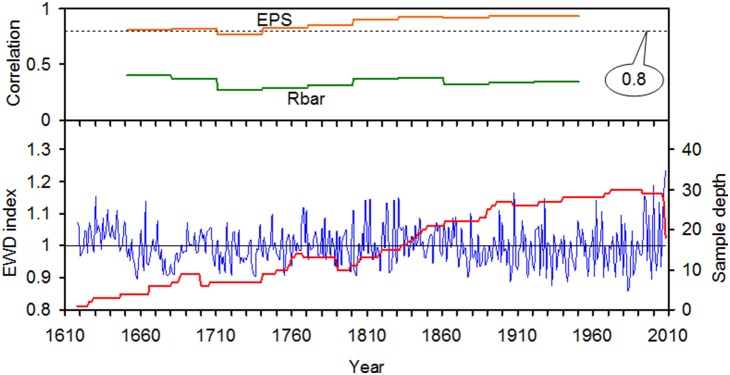
Plot of the standard EWD chronology of the Shimen Mountains, its running expressed population signal (EPS), sample depth and mean inter-series correlation (Rbar).

**Figure 4:**
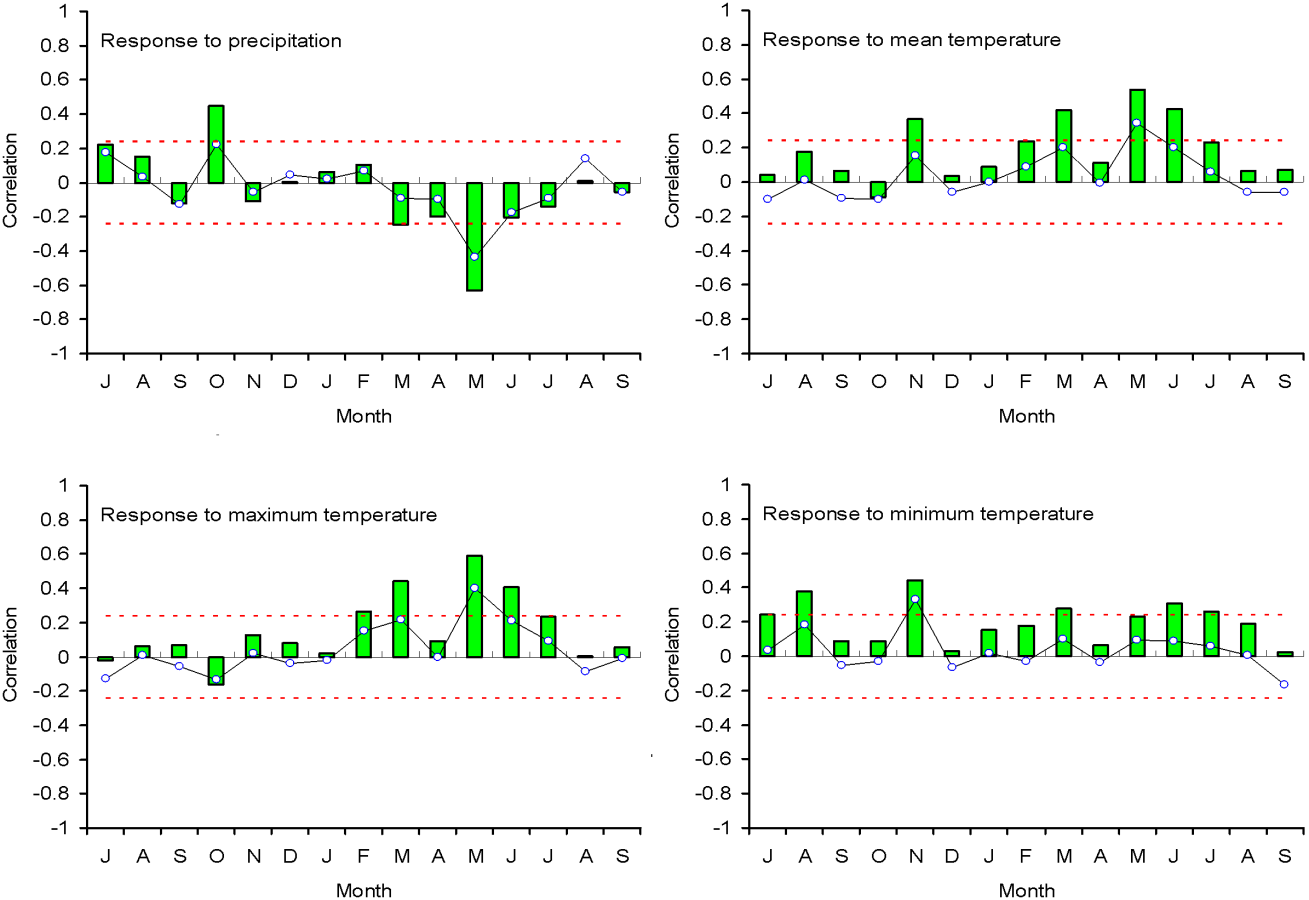
Simple correlations (bars) and response functions (lines) of the standard chronology of the Shimen Mountains with the monthly sum of precipitation and the monthly mean temperature from previous July to current September (1953–2008). The dotted lines indicate significant variables (*p*<0.05).

**Figure 5:**
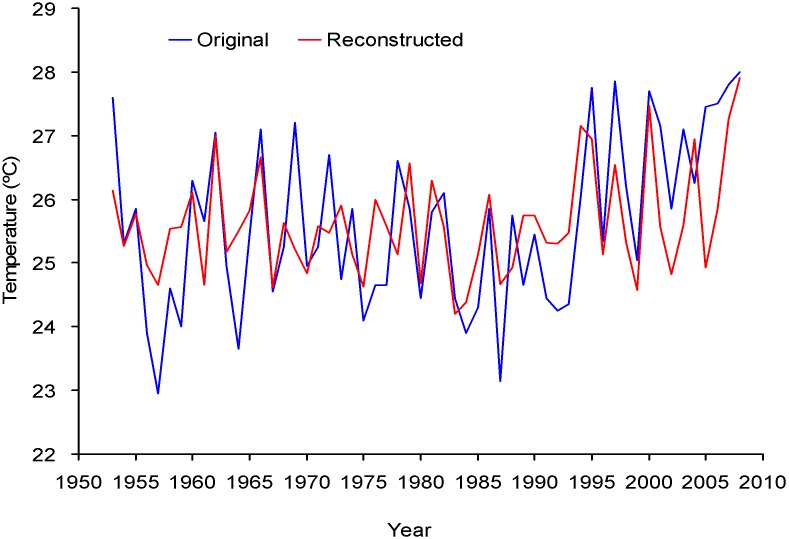
Comparison between observed and estimated spring (May to June) maximum temperature from 1953 to 2008.

As shown in **Figure 3**, a reliable EWD chronology spanning 1666–2008 was developed on the basis of an EPS value greater than 0.8. Although the EPS value of 1711–1740 (0.763) is lower than 0.8, there are still seven series from the four trees, and the EPS value of ring width chronology during this period is higher than 0.9 [33]. Thus, the chronology used in the final reconstruction below was truncated prior to AD 1666 that the retained periods contained at least 4 trees and 6 cores. EWD were negatively correlated with precipitation and positively correlated with temperature (**Figure 4**). Much higher positive correlations were seen between EWD and maximum temperature in current growing season, particularly from May to June. The partial correlation coefficient between EWD and May–June precipitation is −0.28 (*P*<0.05). When precipitation signal was removed by the partial correlation, May–June maximum temperature still have high correlation (*r* = 0.51, *P*<0.01) with EWD. Although EWD was significantly negatively correlated with monthly precipitation, partial correlation results demonstrated that the coincident variation of EWD was controlled by temperature. Therefore, the reconstruction was performed by calibrating the EWD chronology with May–June maximum temperature data. As shown in Table 1, the results of leave-one-out cross-validation showed a good model fit. The reconstruction explained 40.1% of the actual temperature variance during 1953–2008 (**Figure 5**).

**Table 1:** Leave-one-out cross-validation statistics for the May–June maximum temperature reconstruction of Tianshui.

*R*	*r* ^2^	*F*	Signal-test	RE	PMT
0.633	0.401	36.146	38^+^/18^–^	0.396	3.830

### The characteristics of the temperature reconstruction and regional comparison

**Figure 6:**
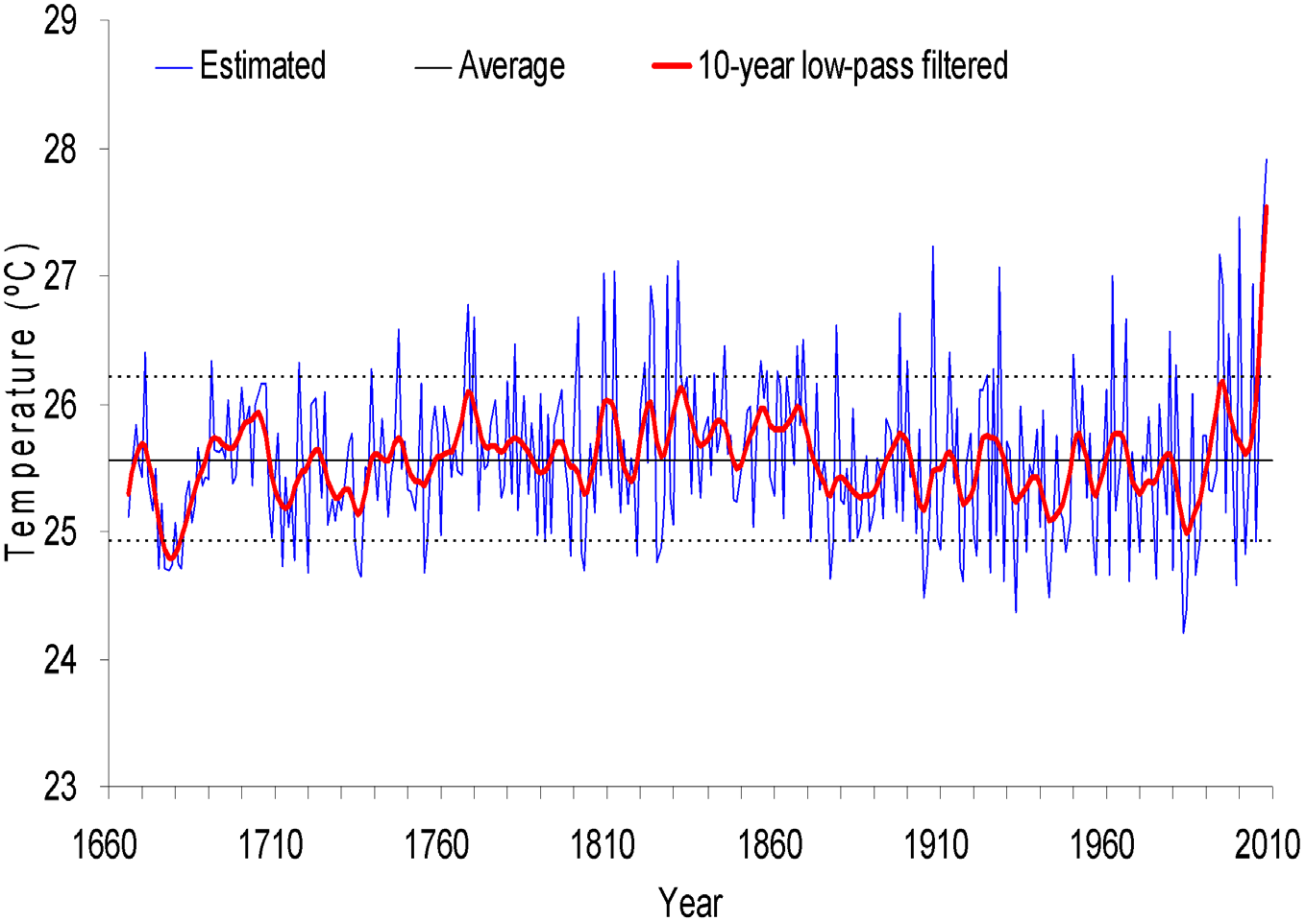
Estimated (thin line) and 10-year low-pass filter (thick line) values of May–June maximum temperature of Tianshui. Central horizontal line shows the mean of the estimated values; The dotted lines show the border of one standard deviation.

The May–June maximum temperature for the 1666–2008 period is 25.6°C, with a standard deviation of σ = 0.4°C. We defined “warm years” as >mean+1σ, and “cold years” were defined as <mean−1σ. During the period of 1666–2008, there are 56 years with cold seasons (16% of the total) and 49 years with warm seasons (14%). The normal years account for 70%. After 10-year low-pass filtered, the temperature reconstruction showed that the cold and warm intervals appear alternatively in decadal scale. This presents eleven cold periods (1666–1689, 1709–1738, 1750–1758, 1788–1792, 1798–1806, 1815–1819, 1872–1894, 1902–1921, 1930–1949, 1955–1959 and 1967–1990), with temperatures lower than the mean; and eleven warm periods (1690–1708, 1739–1749, 1759–1787, 1793–1797, 1807–1814, 1820–1871, 1895–1901, 1922–1929, 1950–1954 and 1991–2008) with temperatures higher than the mean (**Figure 6**).

**Figure 7:**
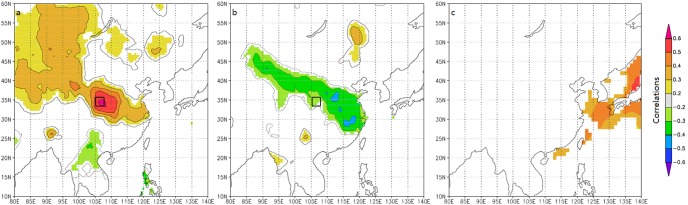
Correlations field maps. (a) Reconstructed May–June maximum temperature of Tianshui and the gridded May–June maximum temperature for the period 1953–2008. (b) Reconstructed May–June maximum temperature of Tianshui and May–June cloud cover for the period 1953–2008. (c) Reconstructed May–June maximum temperature of Tianshui and October–June SSTs for the period 1940–2008.

As shown in **Figure 7a**, significant positive correlations between the temperature reconstruction and the gridded maximum temperature dataset are found with Gansu and Shaanxi, with highest correlations occurring in southeast Gansu. The results confirm that the temperature reconstruction captures the regional temperature signals. Significant (0.1 level) negative correlations with the gridded cloud cover dataset were also found in the same area (**Figure 7b**). Over the common period of 1940–2008, the distinct feature is the significant (0.1 level) positive correlations between temperature reconstruction and SSTs in the western Pacific Ocean (**Figure 7c**).

Based on tree rings of pine trees from Huashan, some tree-ring width and density chronologies were developed [34]. Our EWD chronology is highly correlated with minimum earlywood density chronology of Huashan with *r* = 0.38 (N = 323, *p*<0.001), and the correlation is even lower with *r* = 0.19 (*p*<0.001) after 10-year low-pass filtered. This comparison suggests that earlywood density chronologies mainly contain the high frequency climatic signals. At the same time, the temperature reconstruction are also significantly correlated with the precipitation reconstruction (*r* = −0.52, N = 343, *p*<0.001) [33]. The significant correlation between temperature and precipitation reconstruction is very likely linked with the interactions (*r* = −0.44, N = 56, *p*<0.001) between the actual temperature and precipitation in Tianshui.

### Climate response mechanism

The strong correlation between EWD and spring climate is likely due to moisture limited occurring during the early part of the current growth season. **Figure 2** shows the monthly temperature and precipitation of Tianshui based on long-term averages. The temperature starts rising in April (>10°C), and May–June is a comparatively warm and dry period. Although July and August are the hottest month of the year, moisture limited is no longer a problem because of the ample summer monsoon rainfall. However, during the pre-monsoon and early monsoon season (April–June), the precipitation of Tianshui is low and highly variable. At the warm low elevation sites, such high interannual variability of precipitation could result in decreased soil moisture and increased temperature, leading to high tree growth response.

The EWD of Chinese pine from the Shimen Mountains shows a pattern of responses to climate variables that is opposite to the responses of ring widths [33]. EWD shows significant positive response to May–June temperature and negative response to May precipitation. This is mainly because moisture stress in early stage of growing season suppresses rapid expansion of tracheids [27]. Tree-ring width is the sum of radial diameters of the tracheids, and tracheid diameter contributes to the tree-ring density and width variations. Water deficit in the growing season suppresses enlargement of tracheids. At the same time, evaporation increases with the rise in May–June temperatures due to low precipitation, which accelerates the already existing water stress. When tracheids become narrower, the proportion of cell wall in the annual ring increases because of the reduction of lumen size [35]. This explains the high value of earlywood density in narrow rings (dry years). Essentially, this relationship reflects the short and intense interactions (high frequency signals) between temperature and monsoon rainfall. The high correlations between Huashan chronology and Tianshui chronologies suggest such relationships may exist in a wide range of north central China, and provided the possibility to reconstruct the large scale temperature.

### Linkages with the East Asian summer monsoon

**Figure 8:**
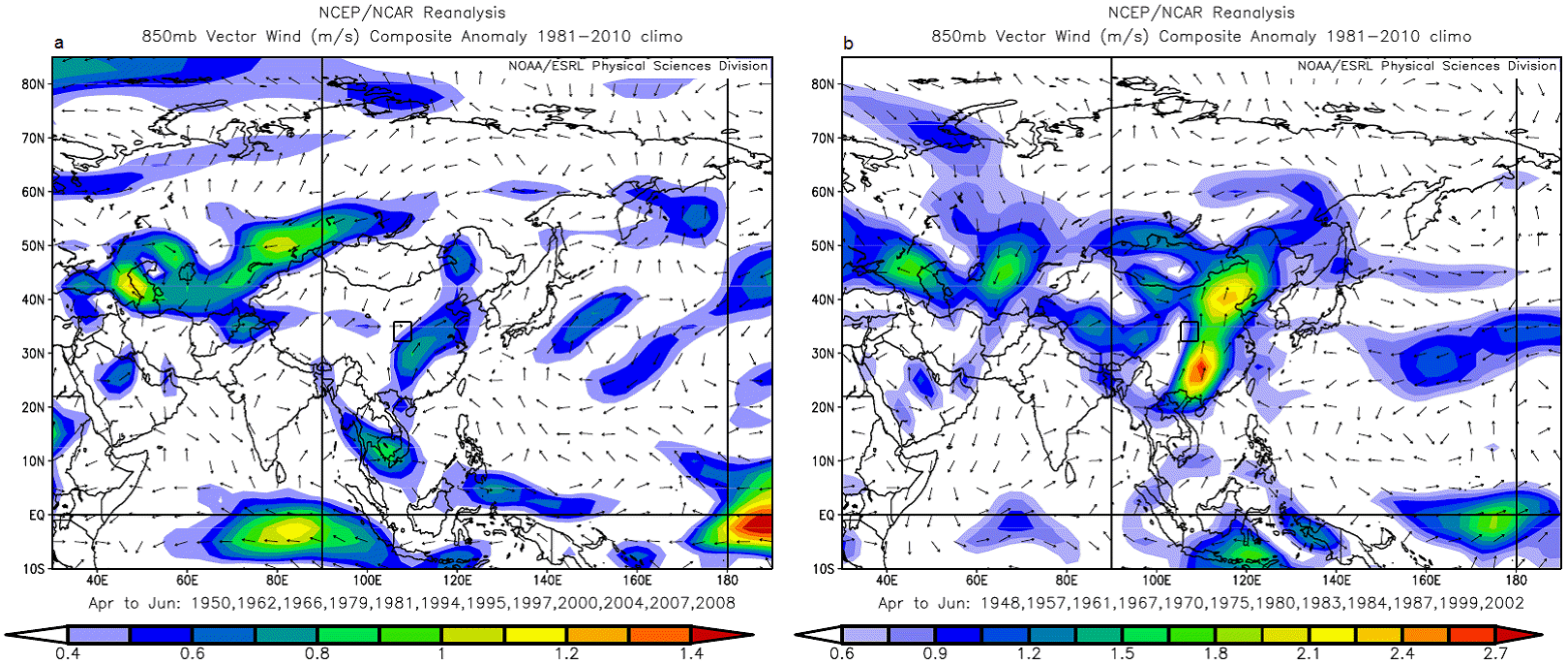
Composite anomaly maps of 850-hPa vector wind for the 12 warmest and 12 coldest years for the reconstructed May–June maximum temperature of Tianshui, 1948–2008. (a) Composite for warmest years (1950, 1962, 1966, 1979, 1981, 1994, 1995, 1997, 2000, 2004, 2007 and 2008). (b) Composite maps for coldest years (1948, 1957, 1961, 1967, 1970, 1975, 1980, 1983, 1984, 1987, 1999 and 2002).

Some tree-ring studies also suggest the possible links of Chinese pines growth in Tianshui with the Asian summer monsoon circulation [34], [36]. The significant positive correlations of the reconstructed temperature with SST in the western Pacific Ocean support the connection with the Asian summer monsoon (**Figure 7**). A positive field is located in the Western North Pacific. When ocean warming was abnormal in this region, the enhanced Western Pacific Subtropical High (warm flow) extended westward, and shifted southeasterly winds to northeasterly winds in north China, and the monsoonal precipitation thus decreased below the normal level [37]–[42]. Consequently, temperature in northern China increased. In contrast, cooling SST of the oceans adjacent to East Asia induced a temperature decrease in north China. The 850-hPa vector wind composite anomaly of the warm years exhibit westerly and northeasterly flow over our study area (**Figure 8a**). Temperature and precipitation composites (not shown) suggest that extremely high reconstructed spring temperature years are characterized by dry and slightly warmer than average conditions. This is consistent with high-level transport of dry air from the Asian continent. In contrast, the 850-hPa vector wind composite anomaly of the cool years exhibit strong southerly and westerly flow over our study area. Temperature and precipitation composites (not shown) suggest that extremely low reconstructed spring temperature years are characterized by wet and slightly cool than average conditions. This is consistent with high-level transport of cool air (Westerly) from the middle-latitude Asian continent, which should provide sufficient cooling to rain out moisture advected from lower latitude sources by the Asian summer monsoon (**Figure 8b**). As discussed above spring temperature conditions in north central China are related to various parts of remote oceans and different aspects of the Asian monsoon. Thus, the scarce rainfall (with the weak monsoon) is the dominant force of spring warming seen in the late 20th century.

## Conclusions

Based on a mean earlywood density chronology of Chinese pine, the spring maximum temperature of Tianshui was reconstructed. The reconstruction explained 40.1% of the variance of the instrumental temperature over the common period 1953–2008. The temperature reconstruction of Tianshui is representative of a large-scale regional temperature variability extending to areas to its southeast and northwest. There existed eleven cold periods (1666–1689, 1709–1738, 1750–1758, 1788–1792, 1798–1806, 1815–1819, 1872–1894, 1902–1921, 1930–1949, 1955–1959 and 1967–1990) and ten warm periods (1690–1708, 1739–1749, 1759–1787, 1793–1797, 1807–1814, 1820–1871, 1895–1901, 1922–1929, 1950–1954 and 1991–2008). There is a high correlation with precipitation reconstruction previously estimated from Tianshui, suggesting a possible connection of regional temperature with the summer monsoon rainfall. This has been supported by significant correlations with SSTs in western Pacific Ocean. Based on analysis of synoptic climatology associated with warm years during the 20th century, we believe that warm springs are characterized by dry conditions and northwesterly upper-level flow over north central China, whereas cool springs are likely characterized by wetter than average conditions brought on by anomalous upper-level flow from the Indian and Pacific Oceans and mean southeasterly flow lower in the troposphere.

This study clearly establishes the potential for using mean earlywood density of Chinese pine from north central China in dendroclimatic studies and brings out the role of mean earlywood density for temperature reconstruction. This increases the options available for reconstructions of climate using tree-ring data over a large area of north central China using longer chronologies.
